# Phyloepigenetics

**DOI:** 10.3390/biology11050754

**Published:** 2022-05-15

**Authors:** Simeon Santourlidis

**Affiliations:** Epigenetics Core Laboratory, Institute of Transplantation Diagnostics and Cell Therapeutics, Medical Faculty, Heinrich-Heine University Duesseldorf, Moorenstr. 5, 40225 Duesseldorf, Germany; simeon.santourlidis@med.uni-duesseldorf.de

**Keywords:** epigenetics, DNA methylation, evolution, phylogenetics, primates

## Abstract

**Simple Summary:**

Epigenetics has established that certain CpG dinucleotides are special genetic spots of supreme functional relevance. It is evident that they are prone to a distinct evolutionary development, compared to other dinucleotides of the genome. Here, the hypothesis is presented that they may be used, in addition to classical phylogenetic analyses, to further dissect species’ relationships.

**Abstract:**

Traditionally, phylogenetic interspecies relationships are estimated based on genetic diversity, since it is assumed that the more recently diverged a species, with comparable constancy of development, the more similar their genetic material and proteins should be. However, occasional controversies in the field may reflect limited resolution and accuracy of this approach. Epigenetics has, meanwhile, provided significant evidence that CpG dinucleotides (CpGs) within genetic material are of particular importance for the annotation and function of the genome and the formation of the phenotype, which is continuously shaped by evolutionary interaction with environmental factors. Based on this, it can be concluded that CpGs follow a distinct rate of evolution, compared to all other nucleotide positions. Evidence is provided that supports this conclusion. Therefore, using CpGs to fathom evolutionary relationships between species could turn out to be a valuable approach to achieve, in some cases, an improved understanding of evolutionary development.

## 1. Introduction

Thanks to C.H. Waddington, we have started to comprehend the fact that epigenetic mechanisms constitute mediating links between the genome and diverse environmental noxae, to which our organism is exposed [1]. Interestingly, these mechanisms feature a certain grade of plasticity, which allows adequate adaptation of the genome’s response, within a defined frame of fluctuation of environmental conditions. In addition, they are heritable from cell to cell, supporting the heritability of the same cell functions and phenotypes. Evidence has risen in the last decades, too, that these features must partly be heritable through the germ line into the next generation, i.e., transgenerational epigenetic inheritance [2]. Gametic epigenetic inheritance [3] refers to the transmission of phenotypic variations to offspring, mediated by epigenetic mechanisms, e.g., DNA methylation. It is established that this mode of inheritance occurs in all taxonomic groups, but the fidelity of its transmission is variable, being condition- and taxon-dependent ([4], and for reviews, see [5,6]). Furthermore, it appears advantageous in randomly and regularly fluctuating conditions [7,8], and contributes to adaptation and distinct dynamics and rates of evolutionary changes in populations. Recent findings on the methylomes of Neanderthals, Denisovans and present-day Homo sapiens have identified around 2000 differential methylated regions (DMRs) in archaic and present-day humans, suggesting epigenetic variations as one important factor driving hominid evolution [9]. This view challenges the currently dominating neo-Darwinian view, and suggests that a significant part of evolution may be epigenetically driven and of distinct plasticity, shaped by fluctuant environmental conditions. This may be responsible for dynamic variations of evolutionary speeds, making evolutionary tracking difficult and blurred. It is an exciting new perspective, closer to which is supposed to be Lamarckian inheritance of acquired characteristics [1]. To understand these dynamics of evolutionary changes in populations, beyond the general patterns of phylogenetic relations, we will need new feasible applications to record epigenetic inheritance.

Traditionally, nucleotide divergence, i.e., interspecies single nucleotide exchanges, also sometimes referred to as single-nucleotide polymorphisms (SNPs), serve as a measurable genetic parameter to estimate phylogenetic relationships of species. This has been further supported by the rapid accumulation of genome sequence data, but phylogenetic uncertainty remains a difficult statistical problem [10]. The coding region of the genome is often preferably considered, which makes up just up to two percent of the whole genetic material, and thus may be of limiting informative value regarding the whole genome. Surprisingly, on this basis, our closest living evolutionary relatives, chimpanzees, have a genome which is almost 99 percent identical to the human one, and 29 percent of genes code for the same amino sequences, where the typical ortholog differs by only two amino acids; one per lineage [11]. This study reports that single-nucleotide substitutions occur at a mean rate of 1.23% between copies of the human and chimpanzee genomes, but, interestingly, CpG dinucleotides substitutions constitute one-quarter of all observed substitutions and occur at more similar rates in male and female germ lines than non-CpG substitutions. Remarkably, these findings uncover that only one change has occurred in a human protein since chimpanzees and humans diverged, about 6.3 million years ago [12]; although there are some conflicting interpretations of ancient fossils of the hominin line dated up to 7.4 million years ago [13,14,15]. Thus, is this genetic difference what makes us humans? Certainly, this is quite a reasonable question of fundamental importance.

A current definition of epigenetics states that epigenetics encompasses all mechanisms which do not act via the DNA sequence itself. These mechanisms modify the DNA and alter chromatin compaction to modulate accessibility for transcription. Heritability of these mechanisms is crucial in this definition to ensure epigenetics-based transmission of characteristics to daughter cells and, on the level of organisms, to their descendants. In addition, more and more it becomes feasible that environmental noxea are appropriately shaping the epigenetic mechanisms over generations, which is why this plasticity should not be neglected within this steadily developing definition of epigenetics, due to accelerated gain of knowledge in this young research field.

The most well investigated epigenetic mechanism is DNA methylation, which occurs at CpG dinucleotides. CpG dinucleotides of the mammalian genome are often situated in regulatory regions of active genes and are generally unmethylated. For instance, more than 60% of human genes possess a 0.4–2 kb long, CpG rich area, surrounding the transcriptional start site, which is called a ‘CpG-island’. In general, these CpG-islands remain unmethylated throughout development and in all tissues, which is thought to protect them from erosion [16]. Methylation of CpG dinucleotides serves as a lock-in mechanism for the transcriptional competence of a genomic region. The methylome, consisting of methylated CpG dinucleotides of the genome of a given cellular state, constitutes an annotating system, defining which parts of the genome will be silent. Methylated CpGs are prone to evolutionary depletion via spontaneous hydrolytic deamination, leading to the replacement of a CpG by TpG, performed by a cellular mismatch repair mechanism. The transition rate of methylated CpG to TpG is 10–50 times higher than other transitional changes [17]. Steadily unmethylated, functionally relevant CpG dinucleotides are exempt from this evolutionary erosion [16].

Therefore, it can be assumed, in analogy to interspecies single nucleotide variations (SNPs), that such functionally relevant CpG dinucleotides should vary lesser between closely related species, which have been diverged recently, as opposed to cases of earlier divergence. Identifying such CpGs and comparing their constancy between related species may provide a new epigenetic parameter to help describe evolutionary relationships with improved accuracy. This approach should better, and more predominantly, work in more closely related species.

It has been suggested, based on the analysis of fossil taxa and on physical, as opposed to genetic, similarities, that orangutans may be humans’ closest relatives, and not chimpanzees [18]. Orangutans share at least 28 unique physical characteristics with us, where chimpanzees possess only two and gorillas seven of them. Furthermore, the authors stated that humans and orangutans share a common ancestor, who had established widespread distribution by at least 13 Ma. and suggested that the divergence of African apes is also older than the oldest fossil members of the dental-hominoid clade—that is, c. 13 Ma. It is inferred that molecular analyses to estimate evolutionary relations are biased by primitive and insufficient retentions, and they can yield phylogenies that are in conflict with traditional taxonomic groupings of primates, leading to their rejection as ‘false’ [12,19].

Of course, given the weight of evidence from the field of genetics, this is a highly provoking view, and, understandably, it is not shared by many scientists in the field. Nevertheless, it should steadily remain in our minds that phylogenetics relies on the assumptions that most recently divergent taxa have to be most similar in their proteins and DNA, and that the pace of molecular change is steady in nature [18].

There exists clear molecular evidence supporting a closer relationship between humans and chimpanzees (e.g., [11,12,19]), and there are also contradictory findings, e.g., massive differences in sequence structure and gene content of the male-specific region of the Y chromosome (MSY) between chimpanzee and human, which are thought to be due to an accelerated evolution during the past six million years [20]. Surprisingly, this study reported that at six million years of separation, the difference in MSY gene content in chimpanzee and human is comparable to the difference in autosomal gene content in chicken and human, at 310 million years of separation.

On the other hand, many researchers suggest the relatively recent split between humans and gorillas of ≤8–10 Ma. [21,22,23]. Gorillas are considered humans’ closest living relatives after chimpanzees, and in 30% of the genome, the gorilla is closer to the human or chimpanzee than the latter are to each other. It has been shown that gorillas share more genes with accelerated rates of evolution with humans than with chimpanzees across a range of significance thresholds [23].

Meanwhile, such controversies may be resolvable, since our recently acquired knowledge from the field of epigenetics on the nature of CpGs suggests an additional approach.

For instance, investigating the consistency of all relevant promoter-associated CpGs within a functionally highly specific gene family, namely Killer Cell Immunoglobulin-like Receptors (KIRs), could provide a new argument in this controversy and may exemplarily indicate that such epigenetically relevant CpGs may serve as epigenetic indicators of species relationships in general. KIRs constitute a main pylon of the innate immune system in defense against infected and transformed cells. The *KIR* locus contains a family of tandemly arrayed, highly homologous, genes, with promoter regions of most *KIR* genes sharing >91% sequence similarity [24]. Small CpG islands are situated within these regulatory regions and epigenetically determine, through DNA methylation and chromatin reorganization, the unique clonotypic expression mode of *KIR* genes in highly specialized Natural Killer Cells (NKs) [25,26,27]. 

Based on *KIR* sequences and haplotypes comparisons across primate species, it has been inferred that *KIR* genes have been predominantly and rapidly evolved in higher primates, perhaps in response to species-specific pathogenic organisms [24]. Thus, analogous to the assumption underlying the phylogenetic based species relations, it can be assumed that the most evolutionarily fast developing, and highly specialized *KIR* genes should coincide with the most recent taxa of higher primates, diverging with the most similar CpG patterns within their epigenetically functional CpG islands.

## 2. Material and Methods

All human *KIR* promoter sequences were derived from the sequence GenBank: AP023479.1. All primate *KIR* sequences were derived from BLAT search on primates, based on human *KIR* promoter sequences from KIR2DL3, KIR2DL1, KIR3DL1, KIR2DS4, KIR3DL2, each of 320 bp length. DNA BLAT at https://genome.ucsc.edu/cgi-bin/hgBlat (accessed on 10 November 2021) [28]. The homologous KIR primate sequences were derived from the assemblies, hg38 (human), gorGor6(gorilla), panTro6 (chimp), panPan3 (bonobo), ponAbe3 (orangutan) and nomLeu3 (gibbon). It has been shown that studies of human, chimpanzee, and orangutan killer cell immunoglobulin-like receptors (KIRs) and their MHC class I ligands provide a model for rapid co-evolution of hominid KIR and MHC genes, and that only the great apes have orthologs encoding all human MHC class I KIR ligands [29].

To align the primate sequences solely on the basis of CpGs an “A” for replacing any CpG in a conserved primate species and a “T” for any CpG mutated in at least one nucleotide in at least one primate species were selected.

All sequences were aligned by PRRN using the default settings and the option progressive (pairwise alignment) and iterative refinement at GenomeNet, https://www.genome.jp/tools-bin/prrn (accessed on 15 November 2021). PRRN uses a hill-climbing algorithm, which optimizes its Multiple Sequence Alignment (MSA) score and iteratively corrects both alignment weights and locally divergent or “gappy” regions of the growing MSA [30]. PRRN is an implementation of the best-first search iterative refinement strategy with tree-dependent partitioning for multiple sequence alignment [31]. The unweighted pair group method was applied with arithmetic mean (UPGMA) as a simple agglomerating (bottom-up) hierarchical clustering method for performing rooted phylogenetic trees [32]. This algorithm is based on the assumption of a molecular clock, i.e., that the taxa are evolving steadily. The *KIR* gene cluster was recently dynamically evolved in primates [24] and provides a model for rapid co-evolution of hominid KIR and MHC genes [29]. This UPGMA is also available by GenomeNet, which is operated using the Supercomputer System of the Institute for Chemical Research, Kyoto University.

## 3. Results

**Figure 1:**
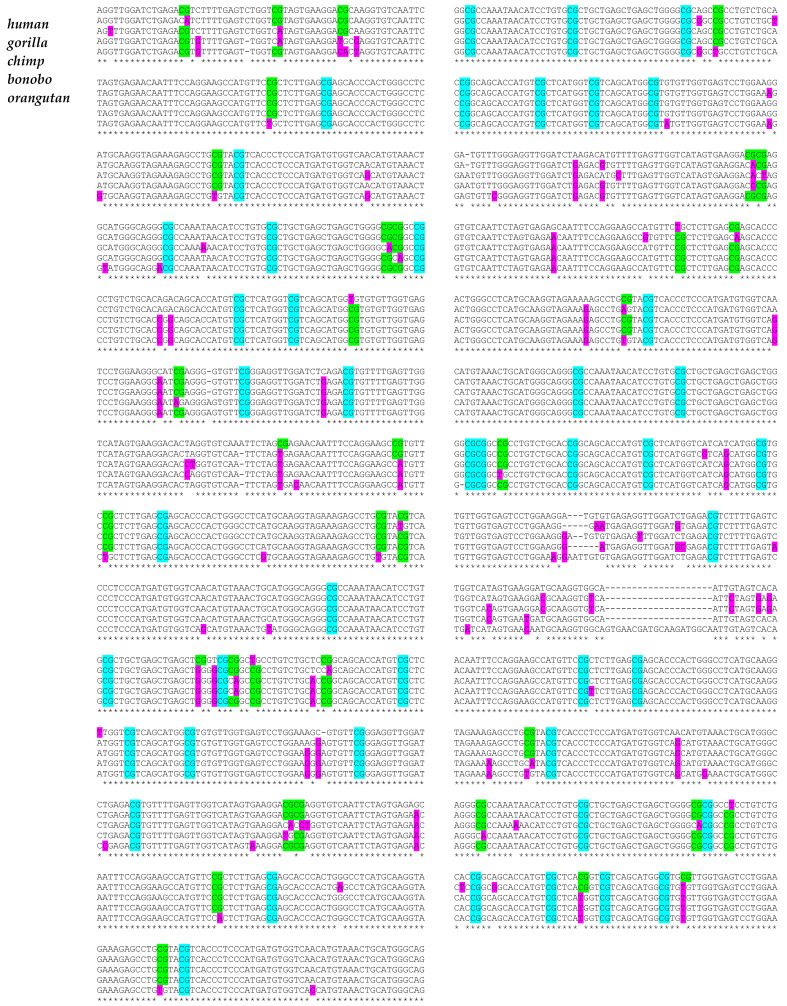
Alignment of assembled *KIR* promoter sequences in hominids. The CpG rich *KIR* gene promoter regions, each of 320 bp length, from the species human, gorilla, chimpanzee, bonobo and orangutan, have been strung together in the order KIR2DL3, KIR2DL1, KIR3DL1, KIR2DS4 and KIR3DL2 and have been aligned. All single nucleotide polymorphisms (SNPs) present in at least one of the five species are highlighted in purple (that is approx. 6.5%). All CpG dinucleotides largely preserved but affected by at least one nucleotide in at least one of the species are highlighted in green (approx. 44% of all preserved CpG positions). All CpG dinucleotides consistently preserved in all species are highlighted in light blue.

**Figure 2:**
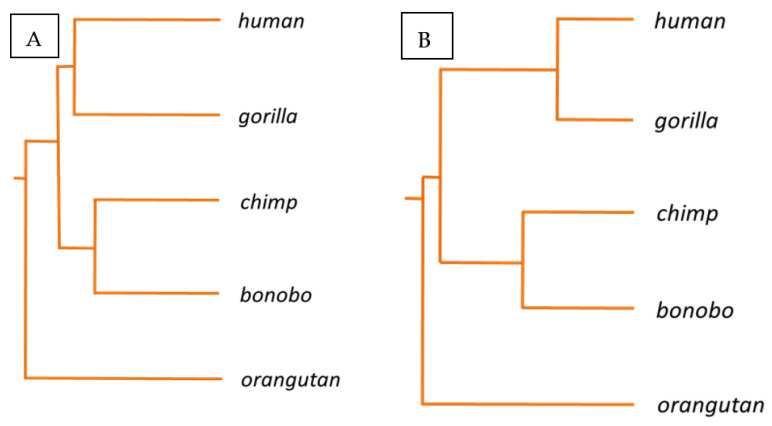
Phylogenetic (**A**) and “phylo-epigenetic” (**B**) relations between 5 primate species based on genetic and epigenetic differences of *KIR* gene promoter regions. The phylogram of the left panel displays the phylogenetic relation of these primate species, based on SNPs. The phylogram of the right panel displays the phylo-epigenetic relation of these species, based on CpG dinucleotide differences of the *KIR* gene promoter regions.

Haplotype A is the main KIR haplotype in humans, consisting of 7 KIR genes strung together. Five of them are clono-typically expressed in subsets of Natural Killer (NK) cells: KIR2DL3, KIR2DL1, KIR3DL1, KIR2DS4 and KIR3DL2. It has been shown that their unique selective expression is governed by differential DNA methylation and chromatin reorganization of the unusual short CpG islands, encompassing their 5′ regulatory regions [24,25,26]. The five *KIR* gene promoter regions from primates—human, gorilla, chimpanzee, bonobo and orangutan—were strung together and aligned. The alignment revealed all interspecies SNPs of these assembled DNA sequences which have been evolutionary accumulated in these primate sequences (Figure 1). Mutations occurred in 6.5% of all nucleotide positions in at least one of these sequences. Based on this alignment the phylogram depicted in Figure 2A situates chimpanzee and bonobo close together, separated shortly after human and gorilla had separated, which also appear relatively closely related, and with the orangutan being the most distant relative.

On the other hand, 44% of all CpG positions, which are largely preserved in all primate sequences, displayed at least one nucleotide change, in at least one of the primate species. Noteworthy, were alterations in one nucleotide of these largely preserved CpGs in at least one of the primates, constituting 2.2% of the whole aligned sequence, in contrast to 0.5%, 0.7% and 0.7%, for AG, TG, GG dinucleotides, respectively. As mentioned above, it has been suggested that this locus should have experienced a dynamic and fast evolution exclusively in primates, due to exposition to comparable immunogenic challenges [24]. Thus, the evolutionary divergence of this epigenetically controlled *KIR* gene system may more sharply mirror recent evolutionary development of these five primate species when examined on epigenetically relevant CpG dinucleotide changes. Solely considering all preserved CpG positions across these five primates, alignments were made, highlighted in green and light blue in Figure 1 (Figure 1). Interestingly, in this case, the phylogram changed slightly. Although the chimpanzee and bonobo remain similarly close together as before, now the human and gorilla appear more closely related than they were before, and they appear to have separated after the chimpanzee and bonobo. Again, the orangutan is the most distant relative (Figure 2B). 

## 4. Discussion

It is established in the field of epigenetics that CpG dinucleotides represent special dinucleotide positions of highly functional relevance for adaptive epigenetic usage of the genome [33]. Therefore, it can be inferred that they should display a distinct evolutionary development, in comparison to other genetic spots, e.g., other mono or dinucleotides. In this study, a certain genetic segment was chosen, in which such functional CpG dinucleotides are periodically organized in front of highly specialized genes of a rapidly involving, functionally specialized genetic area of hominids. A simple statistical analysis revealed that, indeed, the inter-primate divergence is clearer in these CpG dinucleotides than in other comparable dinucleotide positions. In addition, CpG positions were revealed which are consistently unaltered in all 5 primate species, suggesting that they may be of predominant importance for the unique, epigenetically controlled, clonotypic expression mode of *KIR* genes in these species. First, the result of the phylogenetic analysis, based on all SNPs of these regions, resembles the phylogeny among great apes with respect to humans as proposed by Locke et al., where bonobo and chimpanzee are close together, separated ~1 my years ago, followed by human and gorilla, which are thought to have separated ~4–6 and ~6–8 my years ago, respectively. Orangutans separated ~12–16 my years ago [34]. 

However, the phylo-epigenetic result, based on CpG differences, revealed a slightly changed picture of a closer relation between humans and gorillas. It has been suggested that gorillas are humans’ closest living relatives and are of importance for the study of human origins and evolution. This has been underlined by synthesis of genetic and fossil evidence, where, interestingly, 30% of the gorilla genome is closer to humans than to chimpanzees and this is rarer around coding genes, suggesting functional consequences in gene expression [23].

In contrast, it has been suggested by Grehan and Schwartz, based on an array of characters and corroborated by the analysis of fossil taxa, that the African apes are not only less closely related to humans than are orangutans, but also less closely related to humans than are many Miocene hominoids [18].

Based on the results of this paper, the CpG based phylo-epigenetic analyses presented may reflect epigenetic species relationships and may open up a new parameter from the field of epigenetics for supporting, in addition to phylogenetic analysis, the fathoming of evolutionary relationships, presumably just between closely related species. Further similar phylo-epigenetic comparisons over the whole genome are advocated, based on other relevant and functional CpG positions, e.g., CpG islands of housekeeping genes and cell-type specific expressed master regulators of cell identity in embryonic development and/or differentiation. Such research might contribute to improved accuracy and higher resolution in the field of description of evolutionary relationships between all species of fauna and flora. CpG dinucleotides associated with other genes may be as informative as the KIR case and perhaps even more informative when larger CpG islands are analyzed. Thus, gorillas may be the closest relatives to humans, followed by chimpanzees and their relatives, bonobos, with orangutans as the most distant genus. This approach may lead to an improved understanding of the origins and evolution of human beings and the genetic and epigenetic basis of unique human traits and diseases. However, coming back to the similar characteristics between humans and orangutans, i.e., closely related species, it cannot be ruled out that a substantial epigenetic driven convergent evolution of few phenotypic traits may mask the knowledge that is recorded in their origins by an imprecise genetic SNP approach. Extended studies are required to test this *Phylo-epigenetics* approach in respect of its limitations and strengths.

## Data Availability

Data used in this study are duly available from the author on reasonable request.
